# Bis[2-(butyl­imino­meth­yl)-4-chloro­phenolato]iron(II)

**DOI:** 10.1107/S1600536808015080

**Published:** 2008-05-24

**Authors:** Dong-Sheng Xia, Wu Chen, Li-Li Jiang, Qing-Fu Zeng

**Affiliations:** a Engineering Research Center for the Clean Production of Textile Printing, Ministry of Education, Wuhan University of Science & Engineering, Wuhan 430073, People’s Republic of China

## Abstract

In the title compound, [Fe(C_11_H_13_ClNO)_2_], the Fe^II^ atom is four-coordinated in a square-planar geometry by the O and N atoms of two 2-(butyl­imino­meth­yl)-4-chloro­phenolate Schiff base ligands.

## Related literature

For related structures, see: Chen & Wang (2006 ▶); Chen *et al.* (2007 ▶); Ran *et al.* (2006 ▶); Ye *et al.* (2007 ▶); Zhu *et al.* (2003 ▶).
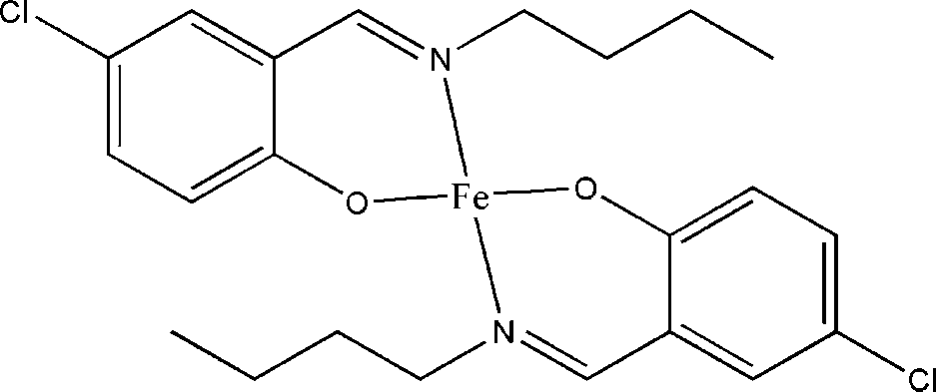


## Experimental

### 

#### Crystal data

[Fe(C_11_H_13_ClNO)_2_]
*M* *_r_* = 477.20
Triclinic, 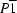

*a* = 10.059 (2) Å
*b* = 10.100 (2) Å
*c* = 11.569 (3) Å
α = 97.093 (3)°
β = 90.800 (2)°
γ = 105.755 (3)°
*V* = 1121.2 (4) Å^3^
*Z* = 2
Mo *K*α radiation
μ = 0.93 mm^−1^
*T* = 298 (2) K
0.32 × 0.32 × 0.28 mm

#### Data collection

Bruker SMART CCD area-detector diffractometer
Absorption correction: multi-scan (*SADABS*; Sheldrick, 1996 ▶) *T*_min_ = 0.755, *T*_max_ = 0.780
4427 measured reflections
4174 independent reflections
3009 reflections with *I* > 2σ(*I*)
*R*_int_ = 0.023

#### Refinement

*R*[*F* ^2^ > 2σ(*F* ^2^)] = 0.065
*wR*(*F* ^2^) = 0.201
*S* = 1.06
4174 reflections
265 parameters
H-atom parameters constrained
Δρ_max_ = 0.91 e Å^−3^
Δρ_min_ = −0.80 e Å^−3^

### 

Data collection: *SMART* (Bruker, 1998 ▶); cell refinement: *SAINT* (Bruker, 1998 ▶); data reduction: *SAINT*; program(s) used to solve structure: *SHELXS97* (Sheldrick, 2008 ▶); program(s) used to refine structure: *SHELXL97* (Sheldrick, 2008 ▶); molecular graphics: *SHELXTL* (Sheldrick, 2008 ▶); software used to prepare material for publication: *SHELXTL*.

## Supplementary Material

Crystal structure: contains datablocks global, I. DOI: 10.1107/S1600536808015080/sj2502sup1.cif

Structure factors: contains datablocks I. DOI: 10.1107/S1600536808015080/sj2502Isup2.hkl

Additional supplementary materials:  crystallographic information; 3D view; checkCIF report

## Figures and Tables

**Table:** 

Fe1—O2	1.890 (4)
Fe1—O1	1.907 (4)
Fe1—N2	2.001 (4)
Fe1—N1	2.010 (4)

**Table:** 

O2—Fe1—O1	176.03 (18)
O2—Fe1—N2	91.69 (17)
O1—Fe1—N2	90.18 (16)
O2—Fe1—N1	87.76 (17)
O1—Fe1—N1	90.95 (16)
N2—Fe1—N1	170.71 (16)

**Fig. 1.**
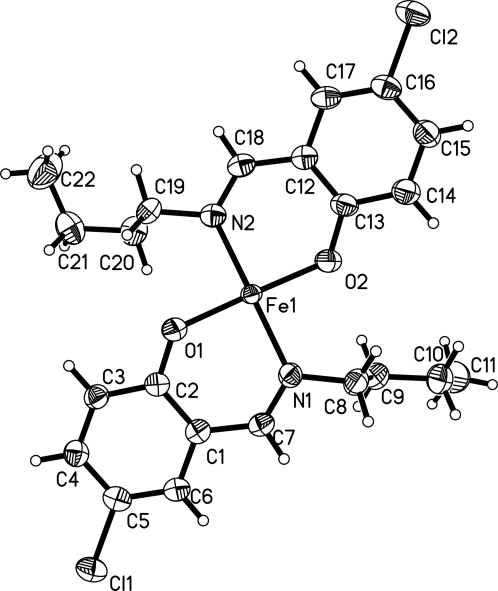
The structure of (I) showing 30% probability displacement ellipsoids and the atom-numbering scheme.

## supplementary crystallographic information

### Comment

As part of our ongoing interest in the structure of iron complexes (Zhu *et al.*, 2003), we report herein the crystal structure of the title compound, a new iron(II) complex, (I), Fig. 1, derived from the Schiff base ligand 2-(butyliminomethyl)-4-chlorophenol.

The Fe^II^ atom in (I) is four-coordinated by two O and two N atoms from two Schiff base ligands, forming a square-planar geometry. The dihedral angle between the two benzene rings is 8.2 (3) °. The coordinate bond distances and angles  (Table 1) are comparable to the values observed in other similar iron(II) complexes (Chen & Wang, 2006; Chen *et al.*, 2007; Ran *et al.*, 2006; Ye *et al.*, 2007).

### Experimental

5-Chlorosalicylaldehyde (31.2 mg, 0.2 mmol), butylamine (14.6 mg, 0.2 mmol), and FeCl_2_ (12.6 mg, 0.1 mmol) were dissolved in methanol (30 ml). The mixture was stirred for 30 min at room temperature in an atmosphere of argon. The resulting solution was left in air for a few days, yielding brown crystals.

### Refinement

H atoms were placed in idealized positions and constrained to ride on their parent atoms with C–H distances in the range 0.93–0.97 Å, and with *U*_iso_(H) set at 1.2*U*_eq_(C) and 1.5*U*_eq_(methyl C).

### Crystal data

**Table:**

[Fe(C_11_H_13_Cl_1_N_1_O_1_)_2_]	*Z* = 2
*M**_r_* = 477.20	*F*_000_ = 496
Triclinic, *P*1	*D*_x_ = 1.414 Mg m^−^^3^
Hall symbol: -P 1	Mo *K*α radiation λ = 0.71073 Å
*a* = 10.059 (2) Å	Cell parameters from 2122 reflections
*b* = 10.100 (2) Å	θ = 2.3–24.5º
*c* = 11.569 (3) Å	µ = 0.93 mm^−^^1^
α = 97.093 (3)º	*T* = 298 (2) K
β = 90.800 (2)º	Block, brown
γ = 105.755 (3)º	0.32 × 0.32 × 0.28 mm
*V* = 1121.2 (4) Å^3^	

### Data collection

**Table:**

Bruker SMART CCD area-detector diffractometer	4174 independent reflections
Radiation source: fine-focus sealed tube	3009 reflections with *I* > 2σ(*I*)
Monochromator: graphite	*R*_int_ = 0.023
*T* = 298(2) K	θ_max_ = 25.5º
ω scans	θ_min_ = 1.8º
Absorption correction: multi-scan(SADABS; Sheldrick, 1996)	*h* = 0→12
*T*_min_ = 0.755, *T*_max_ = 0.781	*k* = −12→11
4427 measured reflections	*l* = −14→14

### Refinement

**Table:**

Refinement on *F*^2^	Hydrogen site location: inferred from neighbouring sites
Least-squares matrix: full	H-atom parameters constrained
*R*[*F*^2^ > 2σ(*F*^2^)] = 0.065	*w* = 1/[σ^2^(*F*_o_^2^) + (0.1206*P*)^2^ + 0.3449*P*] where *P* = (*F*_o_^2^ + 2*F*_c_^2^)/3
*wR*(*F*^2^) = 0.201	(Δ/σ)_max_ < 0.001
*S* = 1.06	Δρ_max_ = 0.91 e Å^−^^3^
4174 reflections	Δρ_min_ = −0.80 e Å^−^^3^
265 parameters	Extinction correction: SHELXL97 (Sheldrick, 2008), Fc^*^=kFc[1+0.001xFc^2^λ^3^/sin(2θ)]^-1/4^
Primary atom site location: structure-invariant direct methods	Extinction coefficient: 0.027 (4)
Secondary atom site location: difference Fourier map	

### Special details

**Table:**

Geometry. All e.s.d.'s (except the e.s.d. in the dihedral angle between two l.s. planes) are estimated using the full covariance matrix. The cell e.s.d.'s are taken into account individually in the estimation of e.s.d.'s in distances, angles and torsion angles; correlations between e.s.d.'s in cell parameters are only used when they are defined by crystal symmetry. An approximate (isotropic) treatment of cell e.s.d.'s is used for estimating e.s.d.'s involving l.s. planes.
Refinement. Refinement of *F*^2^ against ALL reflections. The weighted *R*-factor *wR* and goodness of fit *S* are based on *F*^2^, conventional *R*-factors *R* are based on *F*, with *F* set to zero for negative *F*^2^. The threshold expression of *F*^2^ > σ(*F*^2^) is used only for calculating *R*-factors(gt) *etc*. and is not relevant to the choice of reflections for refinement. *R*-factors based on *F*^2^ are statistically about twice as large as those based on *F*, and *R*- factors based on ALL data will be even larger.

### Fractional atomic coordinates and isotropic or equivalent isotropic displacement parameters (Å^2^)

**Table:**

	*x*	*y*	*z*	*U*_iso_*/*U*_eq_	
Fe1	0.88207 (6)	0.05266 (6)	0.59267 (5)	0.0378 (3)	
Cl1	1.28816 (16)	0.67927 (15)	0.38259 (15)	0.0708 (5)	
Cl2	0.43021 (19)	−0.54688 (18)	0.80626 (17)	0.0875 (6)	
N1	1.0140 (4)	0.2108 (5)	0.6944 (3)	0.0510 (10)	
N2	0.7290 (4)	−0.0862 (4)	0.4948 (4)	0.0505 (10)	
O1	0.9611 (4)	0.1217 (4)	0.4557 (3)	0.0585 (9)	
O2	0.8151 (5)	−0.0192 (4)	0.7305 (3)	0.0742 (12)	
C1	1.0932 (5)	0.3499 (5)	0.5383 (4)	0.0489 (12)	
C2	1.0296 (5)	0.2505 (5)	0.4429 (4)	0.0474 (11)	
C3	1.0436 (6)	0.2901 (6)	0.3317 (5)	0.0546 (13)	
H3	0.9990	0.2270	0.2683	0.065*	
C4	1.1218 (6)	0.4204 (6)	0.3124 (5)	0.0554 (13)	
H4	1.1309	0.4439	0.2371	0.067*	
C5	1.1865 (5)	0.5153 (5)	0.4071 (5)	0.0542 (13)	
C6	1.1748 (5)	0.4835 (5)	0.5187 (5)	0.0508 (12)	
H6	1.2195	0.5484	0.5810	0.061*	
C7	1.0854 (5)	0.3227 (5)	0.6570 (4)	0.0500 (12)	
H7	1.1369	0.3927	0.7128	0.060*	
C8	1.0251 (6)	0.2141 (6)	0.8225 (4)	0.0620 (14)	
H8A	1.1163	0.2703	0.8520	0.074*	
H8B	1.0137	0.1207	0.8410	0.074*	
C9	0.9166 (7)	0.2733 (6)	0.8810 (5)	0.0685 (16)	
H9A	0.9317	0.3682	0.8652	0.082*	
H9B	0.8263	0.2205	0.8469	0.082*	
C10	0.9164 (8)	0.2719 (8)	1.0105 (5)	0.089 (2)	
H10A	1.0081	0.3198	1.0446	0.107*	
H10B	0.8947	0.1766	1.0268	0.107*	
C11	0.8128 (9)	0.3404 (8)	1.0668 (6)	0.103 (3)	
H11A	0.8260	0.4303	1.0423	0.154*	
H11B	0.8259	0.3502	1.1501	0.154*	
H11C	0.7207	0.2842	1.0436	0.154*	
C12	0.6426 (5)	−0.2250 (5)	0.6502 (5)	0.0516 (12)	
C13	0.7240 (6)	−0.1362 (6)	0.7427 (5)	0.0597 (14)	
C14	0.7078 (7)	−0.1759 (7)	0.8555 (6)	0.0753 (18)	
H14	0.7590	−0.1169	0.9183	0.090*	
C15	0.6177 (7)	−0.3004 (6)	0.8748 (5)	0.0718 (17)	
H15	0.6090	−0.3253	0.9497	0.086*	
C16	0.5405 (6)	−0.3874 (6)	0.7816 (6)	0.0632 (15)	
C17	0.5499 (5)	−0.3530 (6)	0.6707 (5)	0.0571 (13)	
H17	0.4963	−0.4126	0.6093	0.069*	
C18	0.6473 (5)	−0.1940 (5)	0.5322 (5)	0.0528 (13)	
H18	0.5860	−0.2570	0.4773	0.063*	
C19	0.7085 (6)	−0.0779 (6)	0.3698 (4)	0.0551 (13)	
H19A	0.6551	−0.1679	0.3313	0.066*	
H19B	0.7976	−0.0547	0.3350	0.066*	
C20	0.6344 (7)	0.0292 (6)	0.3507 (5)	0.0688 (16)	
H20A	0.6864	0.1186	0.3913	0.083*	
H20B	0.5442	0.0044	0.3835	0.083*	
C21	0.6166 (8)	0.0414 (8)	0.2213 (6)	0.084 (2)	
H21A	0.5821	0.1212	0.2149	0.100*	
H21B	0.7070	0.0598	0.1885	0.100*	
C22	0.5237 (12)	−0.0799 (10)	0.1499 (7)	0.132 (4)	
H22A	0.5594	−0.1588	0.1514	0.198*	
H22B	0.5178	−0.0608	0.0711	0.198*	
H22C	0.4334	−0.0993	0.1808	0.198*	

### Atomic displacement parameters (Å^2^)

**Table:**

	*U*^11^	*U*^22^	*U*^33^	*U*^12^	*U*^13^	*U*^23^
Fe1	0.0373 (4)	0.0346 (4)	0.0378 (4)	0.0053 (3)	0.0030 (3)	0.0005 (2)
Cl1	0.0655 (9)	0.0538 (8)	0.0906 (11)	0.0085 (7)	0.0047 (8)	0.0182 (7)
Cl2	0.0835 (12)	0.0614 (10)	0.1079 (13)	−0.0007 (8)	0.0180 (10)	0.0201 (9)
N1	0.046 (2)	0.058 (3)	0.047 (2)	0.013 (2)	0.0061 (18)	0.0053 (19)
N2	0.048 (2)	0.045 (2)	0.059 (3)	0.015 (2)	0.0049 (19)	0.0032 (19)
O1	0.064 (2)	0.050 (2)	0.053 (2)	0.0039 (18)	0.0052 (17)	0.0005 (16)
O2	0.085 (3)	0.059 (2)	0.057 (2)	−0.012 (2)	0.013 (2)	−0.0011 (18)
C1	0.043 (3)	0.052 (3)	0.055 (3)	0.019 (2)	0.002 (2)	0.003 (2)
C2	0.044 (3)	0.046 (3)	0.054 (3)	0.016 (2)	0.001 (2)	0.006 (2)
C3	0.061 (3)	0.049 (3)	0.052 (3)	0.014 (3)	−0.002 (2)	0.004 (2)
C4	0.060 (3)	0.055 (3)	0.056 (3)	0.022 (3)	0.001 (3)	0.012 (2)
C5	0.046 (3)	0.048 (3)	0.074 (4)	0.020 (2)	0.010 (3)	0.010 (3)
C6	0.047 (3)	0.045 (3)	0.061 (3)	0.016 (2)	0.001 (2)	0.002 (2)
C7	0.041 (3)	0.052 (3)	0.055 (3)	0.014 (2)	0.002 (2)	−0.003 (2)
C8	0.060 (3)	0.070 (4)	0.051 (3)	0.012 (3)	−0.002 (3)	0.002 (3)
C9	0.078 (4)	0.062 (4)	0.062 (3)	0.017 (3)	0.003 (3)	0.000 (3)
C10	0.110 (6)	0.088 (5)	0.059 (4)	0.011 (4)	0.009 (4)	0.002 (3)
C11	0.114 (6)	0.098 (6)	0.089 (5)	0.025 (5)	0.036 (5)	−0.012 (4)
C12	0.047 (3)	0.050 (3)	0.059 (3)	0.018 (2)	0.007 (2)	0.002 (2)
C13	0.060 (3)	0.047 (3)	0.065 (3)	0.004 (3)	0.018 (3)	0.002 (3)
C14	0.085 (5)	0.063 (4)	0.065 (4)	0.001 (3)	0.016 (3)	0.002 (3)
C15	0.086 (4)	0.062 (4)	0.065 (4)	0.014 (3)	0.016 (3)	0.013 (3)
C16	0.056 (3)	0.051 (3)	0.082 (4)	0.014 (3)	0.018 (3)	0.011 (3)
C17	0.043 (3)	0.048 (3)	0.077 (4)	0.011 (2)	0.002 (3)	0.001 (3)
C18	0.046 (3)	0.047 (3)	0.064 (3)	0.016 (2)	0.002 (2)	−0.004 (2)
C19	0.057 (3)	0.057 (3)	0.051 (3)	0.021 (3)	−0.004 (2)	−0.005 (2)
C20	0.075 (4)	0.070 (4)	0.070 (4)	0.036 (3)	0.004 (3)	0.004 (3)
C21	0.087 (5)	0.090 (5)	0.091 (5)	0.043 (4)	0.016 (4)	0.034 (4)
C22	0.186 (11)	0.106 (7)	0.102 (7)	0.049 (7)	−0.044 (7)	−0.002 (5)

### Geometric parameters (Å, °)

**Table:**

Fe1—O2	1.890 (4)	C10—C11	1.513 (10)
Fe1—O1	1.907 (4)	C10—H10A	0.9700
Fe1—N2	2.001 (4)	C10—H10B	0.9700
Fe1—N1	2.010 (4)	C11—H11A	0.9600
Cl1—C5	1.753 (5)	C11—H11B	0.9600
Cl2—C16	1.749 (6)	C11—H11C	0.9600
N1—C7	1.291 (7)	C12—C13	1.399 (7)
N1—C8	1.480 (6)	C12—C17	1.422 (7)
N2—C18	1.299 (7)	C12—C18	1.438 (7)
N2—C19	1.474 (6)	C13—C14	1.410 (8)
O1—C2	1.324 (6)	C14—C15	1.381 (8)
O2—C13	1.309 (6)	C14—H14	0.9300
C1—C2	1.412 (7)	C15—C16	1.383 (9)
C1—C6	1.423 (7)	C15—H15	0.9300
C1—C7	1.432 (7)	C16—C17	1.368 (8)
C2—C3	1.392 (7)	C17—H17	0.9300
C3—C4	1.384 (7)	C18—H18	0.9300
C3—H3	0.9300	C19—C20	1.506 (7)
C4—C5	1.388 (8)	C19—H19A	0.9700
C4—H4	0.9300	C19—H19B	0.9700
C5—C6	1.368 (7)	C20—C21	1.529 (8)
C6—H6	0.9300	C20—H20A	0.9700
C7—H7	0.9300	C20—H20B	0.9700
C8—C9	1.510 (8)	C21—C22	1.470 (10)
C8—H8A	0.9700	C21—H21A	0.9700
C8—H8B	0.9700	C21—H21B	0.9700
C9—C10	1.500 (8)	C22—H22A	0.9600
C9—H9A	0.9700	C22—H22B	0.9600
C9—H9B	0.9700	C22—H22C	0.9600
			
O2—Fe1—O1	176.03 (18)	H10A—C10—H10B	107.9
O2—Fe1—N2	91.69 (17)	C10—C11—H11A	109.5
O1—Fe1—N2	90.18 (16)	C10—C11—H11B	109.5
O2—Fe1—N1	87.76 (17)	H11A—C11—H11B	109.5
O1—Fe1—N1	90.95 (16)	C10—C11—H11C	109.5
N2—Fe1—N1	170.71 (16)	H11A—C11—H11C	109.5
C7—N1—C8	114.2 (4)	H11B—C11—H11C	109.5
C7—N1—Fe1	123.9 (4)	C13—C12—C17	120.1 (5)
C8—N1—Fe1	121.6 (3)	C13—C12—C18	123.1 (5)
C18—N2—C19	114.8 (5)	C17—C12—C18	116.8 (5)
C18—N2—Fe1	124.0 (4)	O2—C13—C12	123.9 (5)
C19—N2—Fe1	121.1 (3)	O2—C13—C14	118.1 (5)
C2—O1—Fe1	128.2 (3)	C12—C13—C14	118.0 (5)
C13—O2—Fe1	129.4 (4)	C15—C14—C13	121.6 (6)
C2—C1—C6	120.0 (5)	C15—C14—H14	119.2
C2—C1—C7	123.5 (5)	C13—C14—H14	119.2
C6—C1—C7	116.5 (5)	C14—C15—C16	119.2 (6)
O1—C2—C3	119.6 (4)	C14—C15—H15	120.4
O1—C2—C1	122.4 (5)	C16—C15—H15	120.4
C3—C2—C1	118.0 (5)	C17—C16—C15	121.7 (5)
C4—C3—C2	122.1 (5)	C17—C16—Cl2	119.4 (5)
C4—C3—H3	119.0	C15—C16—Cl2	118.9 (5)
C2—C3—H3	119.0	C16—C17—C12	119.3 (5)
C3—C4—C5	119.0 (5)	C16—C17—H17	120.4
C3—C4—H4	120.5	C12—C17—H17	120.4
C5—C4—H4	120.5	N2—C18—C12	126.2 (5)
C6—C5—C4	121.7 (5)	N2—C18—H18	116.9
C6—C5—Cl1	119.2 (4)	C12—C18—H18	116.9
C4—C5—Cl1	119.0 (4)	N2—C19—C20	111.7 (4)
C5—C6—C1	119.1 (5)	N2—C19—H19A	109.3
C5—C6—H6	120.4	C20—C19—H19A	109.3
C1—C6—H6	120.4	N2—C19—H19B	109.3
N1—C7—C1	126.6 (5)	C20—C19—H19B	109.3
N1—C7—H7	116.7	H19A—C19—H19B	107.9
C1—C7—H7	116.7	C19—C20—C21	112.0 (5)
N1—C8—C9	111.1 (5)	C19—C20—H20A	109.2
N1—C8—H8A	109.4	C21—C20—H20A	109.2
C9—C8—H8A	109.4	C19—C20—H20B	109.2
N1—C8—H8B	109.4	C21—C20—H20B	109.2
C9—C8—H8B	109.4	H20A—C20—H20B	107.9
H8A—C8—H8B	108.0	C22—C21—C20	116.1 (6)
C10—C9—C8	113.9 (6)	C22—C21—H21A	108.3
C10—C9—H9A	108.8	C20—C21—H21A	108.3
C8—C9—H9A	108.8	C22—C21—H21B	108.3
C10—C9—H9B	108.8	C20—C21—H21B	108.3
C8—C9—H9B	108.8	H21A—C21—H21B	107.4
H9A—C9—H9B	107.7	C21—C22—H22A	109.5
C9—C10—C11	112.3 (7)	C21—C22—H22B	109.5
C9—C10—H10A	109.2	H22A—C22—H22B	109.5
C11—C10—H10A	109.2	C21—C22—H22C	109.5
C9—C10—H10B	109.2	H22A—C22—H22C	109.5
C11—C10—H10B	109.2	H22B—C22—H22C	109.5
